# Benefits of Whole-Body Vibration with an Oscillating Platform for People with Multiple Sclerosis: A Systematic Review

**DOI:** 10.1155/2012/274728

**Published:** 2012-05-17

**Authors:** Sebastião David Santos-Filho, Michelle H. Cameron, Mario Bernardo-Filho

**Affiliations:** ^1^Laboratório de Radiofarmácia Experimental, Departamento de Biofísica e Biometria, Instituto de Biologia Roberto Alcantara Gomes, Universidade do Estado do Rio de Janeiro, 20551-030 Vila Isabel, Rio de Janeiro, RJ, Brazil; ^2^Centro de Ciências da Saúde, Universidade Severino Sombra, 27700-000 Vassouras, RJ, Brazil; ^3^Department of Neurology, Multiple Sclerosis Center, Oregon Health & Science University, Portland, OR 97239, USA; ^4^Coordenadoria de Pesquisa, Instituto Nacional do Câncer, 20230-130 Rio de Janeiro, RJ, Brazil

## Abstract

The objective of this work was to investigate the effects of whole-body vibration on people with multiple sclerosis (MS). PubMed, CINAHL and Scopus databases were systematically searched for studies on the use of whole-body vibration (WBV) exercise in people with MS. These searches were supplemented with material identified in the references and in the authors' personal files. A qualitative analysis was performed to summarize the findings. Five studies with a total of seventy-one subjects were identified. All of these studies had small numbers of subjects (3–25), and two of the studies had no control groups. Some investigations have shown significant improvements of the muscle strength, of the functional mobility, and of the timed get up and go test in patients with MS. The number of publications found in the databanks searched is small, and in general, they have limitations in the design of protocols with a weakness to the interpretation of the findings. However, the analysis of the findings in these studies permits to conclude that some papers indicate that WBV exercises could benefit patients with MS. In addition, we suggest further larger scale investigations with controlled parameters and well-designed protocols into the effects of WBV exercises in people with MS.

## 1. Introduction

Multiple sclerosis (MS) is a complex, progressive inflammatory, degenerative, and autoimmune demyelinating disease of the central nervous system (CNS) [1, 2] that causes a wide range of signs and symptoms. The most common signs and symptoms of MS are sensory changes, fatigue, balance disturbances, gait problems, spasticity, motor weakness, and impaired muscular performance [2–4]. Various forms of exercise training have been found to be well tolerated and to improve symptoms in people with MS [5, 6]. Traditionally, these programs have focused on aerobic exercise and resistance training, but, over the last several years, whole-body vibration (WBV) has become increasingly popular as a method of exercise both for people with neurological disorders [7–18] and for the general population [19].

WBV is generated when vibrations produced in an oscillating platform are transferred to a human body or parts of it [20]. It is suggested that the consequences in the body could be due to direct or indirect effects [21]. The indirect effects would be associated with neuroendocrine responses [22]. In the direct effect, muscles and tendons act spring-like elements that store and release mechanical energy, as the vibrations [20]. These facts would induce involuntary muscle contractions that are initiated by sensory receptors and reduce the recruitment threshold of motor units [18]. In consequence some authors have demonstrated an improvement of the ankle plantar flexor strength and power [23] and the enhancement of the stability [24]. In keeping with the WBV working mechanism suggested and the findings reported in the literature [23, 24], it has already been tested in some patients disorders with neurological disorders [7, 8, 17, 25].

As no previous systematic reviews of the effects of WBV exercise on people with MS have been published, the purpose of this study was to systematically review the published research concerning the use of WBV in people with MS.

## 2. Methods

### 2.1. Databanks Used in This Study

PubMed, CINAHL, and Scopus online databases were searched on December 2nd 2010. PubMed comprises more than 20 million citations for biomedical literature from MEDLINE, life science journals, and online books. Citations may include links to full-text content from PubMed Central and publisher web sites [26]. CINAHL is the world's most comprehensive nursing & allied health research database. CINAHL provides indexing for more than 4,500 journals from the fields of nursing and allied health and contains more than 2.7 million records dating back to 1981. CINAHL covers nursing, biomedicine, health sciences librarianship, alternative/complementary medicine, consumer health, and 17 allied health disciplines. Scopus is the world's largest abstract and citation database of peer-reviewed literature and quality web sources. It contains 41 million records, 70% with abstracts, nearly 18,000 titles from 5,000 publishers worldwide, 70% of content is pulled from international sources and includes over 3 million conference papers.

### 2.2. Search Strategy Used to Find the Publications Involving WBV and MS

A search was performed using the keywords: (i) “Multiple sclerosis” and “whole body vibration”, (ii) “Multiple sclerosis” and “whole body vibration exercises”, (iii) “Multiple sclerosis” and “oscillating platform”, and (v) “Multiple sclerosis” and “vibratory platform”.

### 2.3. Inclusion and Exclusion Criteria to Select the Publications

Papers were included for review if they met the search criteria and described a study using whole body vibration generated by an oscillating platform used to treat people with MS and the paper was available in English. Review articles were excluded. These searches were supplemented with material identified in the references and in the authors' personal files. Data were independently abstracted by four of the authors and disagreements were resolved by consensus.

## 3. Results

 The PubMed search yielded 5 publications that met the inclusion criteria of the keywords “multiple sclerosis” and “whole body vibration” and being available in English. A sixth publication with these search terms was available only in Russian. No additional publications were found by searching CINAHL and Scopus or through hand searches of article references. Almost all the publications involving the search with “multiple sclerosis” and vibration were about tests to evaluate somatosensory abnormalities (Table 1).

One publication in Russian [27] was not considered to be discussed in this investigation. The five selected English language publications found with “whole body vibration” and multiple sclerosis were analyzed.

Descriptions of the type of platform, the subjects (number, sex, and age), the frequency and the amplitude used in the oscillating platforms used in these 5 studies are shown in Table 2. It is also shown the use to specify the level of disability of the subjects. Schuhfried et al. [29], Jackson et al. [30], and Broekmans et al. [28] have used the Kurtzke's Expanded Disability Status Scale (EDSS). Wunderer et al. [32] have used the Disease Steps Scale that is a measure of disease progression based on ambulation. Scores range in this scale from 0 (normal) to 6 (confined to wheelchair). Schyns et al. [31] used the Hauser Ambulation Index.

Putting together the information found in the analyzed 5 papers, the numbers of subjects were small ranging from 3 to 25. Moreover, two of the studies had no control groups.

 The study protocols, outcome measures, results, and conclusions drawn by the authors of the 5 selected papers are summarized in Table 3. Outcome measures of functional ambulation, balance, lower extremity strength, well being and spasticity were determined with different tests, as timed get up and go test (TUGT) (functional mobility) [28, 29, 31, 32], functional reach test [29], isometric quadriceps and hamstring muscle torque [30], ten-metre walk, modified ashworth scale, multiple sclerosis spasticity scale (MSSS-88), lower limb muscle force, nottingham sensory assessment and multiple sclerosis impact scale (MSIS-29) [31], strength of the ankle plantar flexors and knee extensors (Nicholas manual muscle tester) [32], knee-muscle maximal isometric and dynamic strength, strength endurance and speed of movement (isokinetic dynamometry), Berg balance scale, two-minute walk Test, and the timed 25-foot walk test (Function) [28].

Schuhfried et al. [29] reported a strong effect of the WBV in one week after the intervention, where significant differences for the change score were found for the TUGT. However, no differences were found for the Functional Reach Test.

The study of Jackson et al. [30] had no control group. They compared the effects of two different frequencies of WBV, 2 and 26 Hz, on lower extremity muscle strength and found no significant differences in isometric torque production between these two frequencies. Although not statistically significant, peak torque values for both quadriceps and hamstring muscles were consistently higher after 30 seconds of WBV at 26 than at 2 Hz.

Schyns et al. [31] found that exercise program had positive effects on muscle force and wellbeing, but the addition of WBV did not appear to provide any additional benefit. The Modified Ashworth Scale was generally unaffected, although, for each group, results from the MSSS-88 showed WBV and exercises reduced muscle spasticity. Results for the 10-m walk and TUGT improved, but without statistical significance.

The study of Wunderer et al. [32] also had no control group and had only 3 subjects. In all subjects plantar flexor strength improved significantly after WBV and in two subjects had significantly in functional mobility as measured by TUGT.

Broekmans et al. [28] found that lower extremity muscle performance and functional capacity were not altered following 10 or 20 weeks of WBV.

## 4. Discussion

Although, WBV is widely available to exercisers and patients, it seems that this modality is still unknown to the scientific community [20]. Concerning to the use of WBV in patients with MS, the number of publications found in the databanks, as PubMed, CINAHL and Scopus, is too small. Some authors have reported significant positive effects of the WBV [29, 32], other authors have found improvements that were not significant [31] in patients with MS, while other authors have not found improvement in these patients [28]. In another paper [30], the effect of two frequencies used in the oscillating platform were studied and, although without significant difference, a consistent trend of higher torque values was found with 26 Hz in comparison with 2 Hz. The most important question in the studies was the small number of subjects that were evaluated in the studies, from three [32] up to 25 [28]. Furthermore, in two papers that was not a control (placebo) group [30, 31]. In addition, in a revision describing clinical findings involving the use of WBV exercises, Rittweger [20] has discussed that, in patients with Parkinson's disease, acute WBV (frequency = 6 Hz, amplitude = 1.5 mm, random) has been found to reduce body sway and to reduce tremor and rigidity. Moreover, Rittweger described that, very similarly, acute WBV (frequency ≥ 1 Hz, amplitude = 3 mm, random) has been found to reduce body sway and the TUGT time in patients with MS. An important feeling in this revision that we agree is that although these studies only involved small sample sizes and only investigated acute effects, they may constitute first hints for the efficacy of WBV in progressive neurological of diseases related with the CNS.

As Rittweger [20], Madou and Cronin [33] have also reviewed the effects of WBV on physical and physiological capability in special populations with subjects with also neurological conditions. The average PEDro score was 4.93 (±1.59). With 60-second intervention and 60-second rest periods, the most frequent vibratory stimulation loading parameters used were 3–6 Hz and 3 mm amplitude for MS and Parkinson's disease patients and 30 Hz and 3–5 mm amplitude for all other conditions. Balance, stability, and functional performance significantly improved in all special population WBV intervention groups as compared with the control groups. Bone mass density and isometric leg strength improvements were also reported. Schuhfried et al. [29], Jackson et al. [30], Schyns et al. [31], and Wunderer et al. [32] have also reported some improvement of the clinical approaches of the patients with MS submitted to the WBV intervention.

In addition, in general, exercise therapy has been considered to be an important and supportive treatment for people with neurological disorders [34, 35], as MS [36]. However, the number of publication involving MS and exercises is still limited (see Table 3). A Cochrane systematic review [36] exploring the effects of exercise for people with MS showed that exercises have beneficial effects on strength, physical endurance, mobility-related activities (transfer, balance, and walking) and on mood, without any evidence of detrimental effects. WBV exercises are performed in oscillating platforms, and Madou and Cronin [33] have reviewed the effects of WBV on physical and physiological capability in special populations and they concluded that WBV provides alternative and/or additional therapeutic interventions to improve physical and functional performance. The specific loading parameters and the value of WBV as compared with conventional interventions need to be the source of future research.

MS is associated with multiple impairments of muscle, sensation, coordination, and balance [37]. In consequence, these conditions can lead to a decrease in physical activity [38]. It would be expected that WBV exercises would seem an important alternative to the management of patients with MS due to some benefits related to the action in the muscle performance [11, 14] and in the some neurological functions [13].

Of the 5 publications on WBV for people with MS found in the literature, all were limited by small sample size and two had no control group.

Two of the studies reported significant benefits, one reported a trend towards but not a statistically significant benefit and a fourth found no benefits. The fifth publication [30] on this topic compared two different frequencies of WBV for people with MS and found no significant difference between baseline torque values and those measured at one, 10, and 20 minutes after either vibration exposure. However, there was a consistent trend of higher torque values after the 26-Hz WBV when compared with the 2 Hz condition for both quadriceps and hamstring muscles. Putting together the results reported by the authors, probably the different findings might be related with the parameters used in the oscillating platform, as the amplitude and the frequency, the design of protocols used, and the individual characteristics of the patients. Moreover, the number of subjects that has been utilized in the works is too small and in addition, the time of the protocols of WBV exercise may have too short. A general analysis of the publications reveals a weakness of the published papers.

Considering the TUGT, four of the five studies have used this test. Broekmans et al. [28] have observed that, at baseline, there were no statistically differences between the groups. Wunderer et al. [32] have reported that a subject improved significantly in the TUGT and the improvements were also clinically significant with a mean decrease in time to perform the TUGT of 18%. Schyns et al. [31] have described that, although results for the TUGT improved, this did not reach statistical significance. Schuhfried et al. [29] for the TUGT, all measurements after the intervention tended to result in better (lower) values for the WBV group compared with the placebo group. In the examination one week after the intervention a significant difference in the change score in favour of the WBV was found. Moreover, two weeks after the intervention the values for TUGT in the WBV group were still higher than in the placebo group, however, without reaching statistical significance for the change score.

In conclusion, the number of publications found in the databanks searched involving WBV and MS is small, and, in general, they have limitations in the design of protocols with a weakness to the interpretation of the findings. The analysis of the findings of the studies indicates that the WBV could bring some benefits to the patients with MS due to in different populations generated significant improvements in a wide variety of muscle strength and functional parameters. Although, positive effects have been reported, only one of the studies analyzed in this work was a randomized controlled trial.

In addition, we suggest further larger scale investigations with controlled parameters and well-designed protocols into the effects of WBV exercises in people with MS. This would be highly desired to try to improve the quality of life of the patients with this disease.

## Figures and Tables

**Table 1:** Publications involving multiple sclerosis and some neurological.

Keywords searched	Number of publications
“Multiple sclerosis”	44520
“Multiple sclerosis” and exercise	446
“Multiple sclerosis” and vibration	67
“Multiple sclerosis” and “whole body vibration”	6 [27–32]
“Multiple sclerosis” and “whole body vibration exercises”	No items found
“Multiple sclerosis” and “oscillating platform”	No items found.
“Multiple sclerosis” and “vibratory platform”.	No items found.

**Table 2:** Data about the devices of the oscillating platform, the subjects, the frequency, and the amplitude used in the oscillating platforms.

Reference	Subjects (sex, age, groups)	Platform manufacturer	Oscillation frequency/amplitude	Disability level of the subjects
Schuhfried et al., [29]	Two groups: intervention (1 male/5 female, 49.3 y) and placebo (2 male/4 female, 46.0 y)	Zeptor: Med system (Scisen GmbH, Germany)	From 1 Hz/3 mm until the patient does not tolerate a further increase.	Subjects with an impairment of ≤5 based on Kurtzke's expanded disability status scale (EDSS).
Jackson et al., [30]	15 subjects (3 male/12 female, 54.6 y) were divided in two groups. A group was submitted to 2 Hz and the other one was submitted to 26 Hz	Maxuvibe platform (Fitgroup BV, Hoogstraat, Holland)	2 or 26 Hz/6 mm	Subjects with an impairment of ≤6.5 based on Kurtzke's EDSS.
Schyns et al., [31]	Two groups: Group I (5 females/3 males, 45.8 years old) and Group II (7 females/1 male, 49.5 years old)	VibroGym International BV, The Netherlands	40 Hz/2 mm	Subjects with an impairment between 1 and 6 on the Hauser ambulation index
Wunderer et al., [32]	3 subjects	VibroGym apparatus (Professional model, ES Haarlem, The Netherlands)	40 Hz/2 mm	Scores in the disease steps scale of the subjects were 2 with abnormal gait, no need for walking aid, 4 depend on unilateral support, and 5 need for bilateral support.
Broekmans et al., [28]	Two groups: WBV group (7 females/4 males, 46.1 years old) and Control group (11 females/3 male, 49.7 years old)	Alpha Vibe Nijverdal, The Netherlands	25–45 Hz/2.5 mm	Subjects with an EDSS score ranging from 1.5 to 6.5

**Table 3:** Study protocols, measures, results, and conclusions from the selected papers.

Reference	Study protocols	Measures	Results	Conclusion
Schuhfried et al., [29]	Beginning with 1 Hz increasing until the patient does not tolerate. With this frequency 5 series of 1 min each with breaks of 1 min each was done. In the placebo group a Burst-TENS application on the nondominant forearm in 5 series of 1 min each with a 1 min break between the series.	Posturographic assessment using the sensory organization test and the TUGT at each time point of measurement after the application.	Compared with the placebo group, the intervention group showed advantages.	Conclusion: the results of this pilot study indicated that WBV may positively influence the postural control and mobility in MS patients.
Jackson et al., [30]	After baseline measures of IT (quadriceps and hamstring muscle), subjects received WBV either 2 or 26 Hz.	Torque values were measured again at one, 10, and 20 minutes after vibration.	No significant differences in IT between 2 and 26 Hz. But, there was a consistent trend of higher torque values after the 26 Hz WBV when compared with the 2 Hz for quadriceps and hamstring muscles.	Whether WBV presents a viable treatment option as either a warm-up activity or a long-term exercise intervention is yet to be determined.
Schyns et al., [31]	Group treated: 4 weeks of a set exercise with WBV (40 Hz, 30 s), 3 times per week, followed by a rest period of 2 weeks and a further 4-week period of the same exercises without WBV, 3 times per week. Group control: exercise without WBV for 4 weeks first, rest for 2 weeks, and 4 weeks of exercise and WBV.	Ten-metre walk, TUGT, modified ashworth scale, multiple sclerosis spasticity scale (MSSS-88), lower limb muscle force, Nottingham sensory assessment, and MS impact scale (MSIS-29) were used before and after intervention.	Exercise program improved muscle force and wellbeing, but there the addition of WBV provided no further benefit. The 10 m walk and TUGT improved but without statistical significance. For most subjects sensation was unaffected by WBV.	Exercise may be beneficial to those with MS, but there is limited evidence that the addition of WBV provides any additional improvements.
Wunderer et al., [32]	Procedure included a 4-week baseline phase without intervention, 6 weeks of twice weekly WBV (40 Hz) on a platform, and a 4-week baseline phase without intervention. A single subject experimental design was replicated on three subjects.	During all phases, strength of the ankle plantar flexors and knee extensors was assessed twice weekly with the Nicholas manual muscle tester and functional mobility with the TUGT.	All subjects improved significantly in PFS. One subject improved significantly in KES bilaterally and one subject in the weaker leg. Two subjects improved significantly in functional mobility. Improvements in strength and mobility were maintained in the final baseline phase.	Regular WBV training can improve lower limb strength and mobility in some individuals with MS.
Broekmans et al., [28]	WBV group performed static and dynamic leg squats and lunges on a vibration platform during 20 weeks (5 training sessions per 2-week cycle). Control group maintained their usual lifestyle.	PRE-, MID- (10 weeks), and POST- (20 weeks) knee-muscle maximal isometric and dynamic strength, strength endurance and speed of movement were measured. Function was determined through the Berg balance scale, TUGT, two-minute walk test and the timed 25-foot walk test.	Leg muscle performance and functional capacity were not altered following 10 or 20 weeks of WBV.	Under the conditions of the present study, the applied 20-week WBV exercise protocol did not improve leg muscle performance or functional capacity in mild-to-moderately impaired persons with MS during and immediately after the training program.

TENS—transcutaneous electrical nerve stimulation

TUGT—Timed Up and Go Test

IT—isometric torque

PFS—plantar flexor strength

KES—knee extensor strength.
